# Supporting Expectant and Parenting Teens: New Evidence to Inform Future Programming and Research

**DOI:** 10.1007/s10995-020-02996-2

**Published:** 2020-08-29

**Authors:** Jessica F. Harding, Susan Zief, Amy Farb, Amy Margolis

**Affiliations:** 1 Mathematica, P.O. Box 2393, Princeton, NJ 08543-2393 USA; 2 Office of Population Affairs, Washington, DC USA

**Keywords:** Teen parents, Young parents, Pregnancy Assistance Fund, Implementation, Evidence, Effectiveness, Recruitment, Partnerships, Sustainability, Teen mothers, Teen fathers, Teen moms, Teen dads

## Abstract

Until recently, federal programs had not explicitly focused on improving the outcomes of highly vulnerable teen parents. Established in 2010, the Pregnancy Assistance Fund (PAF) aims to improve the health, social, educational, and economic outcomes for expectant and parenting teens and young adults, their children, and their families, through providing grants to states and tribes. This article introduces the *Maternal and Child Health Journal* supplement “Supporting Expectant and Parenting Teens: The Pregnancy Assistance Fund,” which draws together the perspectives of researchers and practitioners to provide insights into serving expectant and parenting teens through the PAF program. The articles in the supplement include examples of programs that use different intervention strategies to support teen parents, with programs based in high school, college, and community settings in both urban and rural locations. Some of the articles provide rigorous evidence of what works to support teen parents. In addition, the articles demonstrate key lessons learned from implementation, including allowing some flexibility in implementation while clearly outlining core programmatic components, using partnerships to meet the multifaceted needs of young parents, hiring the right staff and providing extensive training, using strategies for engaging and recruiting teen parents, and planning for sustainability early. The studies use a range of qualitative and quantitative methods to evaluate programs to support teen parents, and three articles describe how to implement innovative and cost effective methods to evaluate these kinds of programs. By summarizing findings across the supplement, we increase understanding of what is known about serving expectant and parenting teens and point to next steps for future research.

## Significance Statement

There is limited research on what works to support expectant and parenting teens and how to successfully implement programs to serve them. This introductory article highlights key findings and lessons learned from the supplement “Supporting Expectant and Parenting Teens: The Pregnancy Assistance Fund,” which provides insights into serving expectant and parenting teens through the Pregnancy Assistance Fund. Rigorous research shows programs can improve outcomes for teen parents. Key lessons learned from program implementation include allowing some flexibility in implementation while clearly outlining core programmatic components, using partnerships to meet the multifaceted needs of young parents, hiring the right staff and providing extensive training, using strategies for engaging and recruiting teen parents, and planning for sustainability.

## Introduction

Expectant and parenting teens face immense challenges completing education, finding work, and earning family-supporting wages (Assini-Meytin and Green 2015; Diaz and Field 2016; Fletcher and Wolfe 2009, 2012; Lee 2010). Nearly one in every five births to teens is a repeat birth (Centers for Disease Control and Prevention 2013), which can compound the challenges teen parents face (Jones and Mondy 1994; Klerman 2004). Until recently, federal programs had not explicitly focused on a clear programmatic gap—supporting highly vulnerable teen parents. Established in 2010, the Pregnancy Assistance Fund (PAF) program aims to improve the health, social, educational, and economic outcomes for expectant and parenting teens and young adults, their children, and their families (Patient Protection and Affordable Care Act 2010). Through the Office of Population Affairs, PAF provides grants to states and tribes (referred to as “PAF grantees”) to support teen parents and their families. PAF grantees use grant funds to establish, maintain, and/or operate expectant and parenting student services in high schools, community service centers, and/or institutions of higher education; improve services for pregnant women who are victims of violence; and increase awareness of available services and resources for expectant and parenting young families (for more information about PAF, see Margolis et al. 2020, this issue).

This article introduces the *Maternal and Child Health Journal* supplement “Supporting Expectant and Parenting Teens: The Pregnancy Assistance Fund,” which draws together the perspectives of researchers and practitioners to provide insights into the PAF program. The articles in the supplement include examples of programs that use different intervention strategies to support teen parents, with programs based in high school, college, and community settings in both urban and rural locations. Some of the articles provide rigorous evidence of what works to support teen parents. In addition, the articles demonstrate key lessons learned from implementation, including allowing some flexibility in implementation while clearly outlining core programmatic components, using partnerships to meet the multifaceted needs of young parents, hiring the right staff and providing extensive training, using strategies for engaging and recruiting teen parents, and planning for sustainability early. The studies use a range of qualitative and quantitative methods to evaluate programs to support teen parents, and three articles describe how to implement innovative and cost effective methods to evaluate these kinds of programs^1^. By summarizing findings across the supplement, we increase understanding of what is known about serving expectant and parenting teens and point to next steps for future research.

## Programs Can Improve Outcomes for Teen Parents

The evidence in this supplement demonstrates that programs can improve aspects of teen parents’ self-sufficiency. Specifically, a systematic review found 14 programs with rigorous evidence of improving teen parents’ education, increasing contraceptive use, or reducing repeat pregnancies (Harding et al. 2020, this issue). Consistent with prior work (Steinka-Fry et al. 2013), this systematic review found that programs can improve teens’ educational outcomes, particularly outcomes related to educational progress such as school enrollment (Harding et al. 2020, this issue). Supporting this conclusion are findings from a rigorous quasi-experimental study of New Heights, a school-based program to support teen parents, which demonstrated improvements in teen parents’ educational outcomes (Asheer et al. 2017b; Zief et al. 2020a, this issue). Another important outcome for teen parents is contraceptive use, which supports healthy birth spacing (Bennett et al. 2006; Raneri and Wiemann 2007; Stevens-Simon et al. 2001; Coard et al. 2000). As far as we are aware, the systematic review in this supplement was the first to examine whether programs can impact teen parents’ contraceptive use (Harding et al. 2020, this issue). The systematic review found that some programs increased contraceptive use, with some effects sustained more than 9 months after the program ended. These results are supported by findings from a well-conducted randomized controlled trial of Healthy Families Healthy Futures enhanced with Steps to Success, a home visiting program for teen parents, which found suggestive evidence of increases in use of long-acting reversible contraceptives among the treatment group (Zief et al. 2020b, this issue). Finally, consistent with other research (Corcoran and Pillai 2007), the systematic review found programs reduced repeat pregnancies or births, particularly rapid repeat pregnancies or births that occurred within 24 months of prior births (Harding et al. 2020, this issue). Overall, the supplement provides rigorous research about programs that promote teen parents’ education, increase contraceptive use, or reduce repeat pregnancies. Improving these outcomes can support teen parents’ self-sufficiency and ability to support their children (Assini-Meytin and Green 2015; Bjerk 2012; Campbell 2015; Diaz and Field 2016; Jones and Mondy 1994; Klerman 2004; Lee 2010; Oreopolous 2008).

## Diverse and Flexible Program Models Can Support Teen Parents

The PAF program allows states and tribes to use varied, multi-component models so they can tailor to the needs of their expectant and parenting teens (Margolis et al. 2020, this issue). This is consistent with research suggesting that different kinds of models can promote positive outcomes. For example, the effective programs identified in the systematic review used different intervention strategies, although most provided intensive one-on-one support to teen parents (Harding et al. 2020, this issue). Similarly, the two programs that demonstrated impacts on teen parents’ outcomes included in this supplement used different approaches to providing one-on-one support: New Heights was a school-based program that included case management (Asheer et al. 2020b, this issue) and Healthy Families Healthy Futures enhanced with Steps to Success was based on a home visiting model (Zief et al. 2020b, this issue). The other programs described in this supplement provided school- or college-based supports (Aufrichtig et al. 2020, this issue; Askelson et al. 2020, this issue; Amenumey et al. 2020, this issue), provided case management (Asheer et al. 2020a this issue; Kang et al. 2020, this issue; Egan et al. 2020, this issue; McGirr et al. 2020, this issue), or focused on systems-level coordination among service providers (Purington et al. 2020a, this issue; Workman and Browder 2020, this issue). Taken together, these studies illustrate that effective programs can use different strategies—there is no one size fits all approach to serving teen parents.

Even within particular intervention strategies or program models, the articles in this supplement suggest that allowing some flexibility to tailor to different contexts can be useful. For example, two studies of school-based programs that were widely implemented on a large scale described how having core programmatic components while allowing flexibility with the program model enabled sites to tailor to their population (Asheer et al. 2020b, this issue; Aufrichtig et al. 2020, this issue). The New Mexico Graduation Reality And Dual-role Skills (GRADS) program is a statewide program that provides a classroom-based course, case management, and health services as well as additional services such as child care and father-focused case management. Schools have flexibility to determine which additional services to provide based on their context and available services within their districts (Aufrichtig et al. 2020, this issue). Program leaders considered this flexibility important for fostering buy-in from school and district leaders. Similarly, New Heights was implemented in schools across Washington, DC, and offered students case management, advocacy, education, and in-kind incentives. School-based coordinators could tailor services to the needs of students in each particular school (Asheer et al. 2020b, this issue). In contrast, when the statewide Adolescent Family Life Project-Positive Youth Development program implemented more consistency in content and approach across numerous sites in California, a study of its early implementation found that staff perceived that more flexibility was necessary to meet the specific needs of the mothers they served (Asheer et al. 2020a, this issue). A key lesson drawn from these studies is that program developers and supervisors should incorporate guidance on how and when programs can be flexible while maintaining fidelity to what they consider as core components.

## Partnerships are Useful for Meeting the Multifaceted Needs of Young Parents

Expectant and parenting teens have complex and interconnected needs (Pinzon and Jones 2012; Savio Beers and Hollo 2009). They need services to support their health, the health of their child, their educational attainment, their employment, positive parenting, and healthy relationships. They also need concrete supports such as child care, baby supplies, food, and safe housing. Given the complex nature of these needs, no single organization is likely to be able to meet them by itself. Consistent with this, PAF grantees created many partnerships. The 19 grantees in the 2017–2018 PAF cohort collaborated with 2042 partners, such as educational institutions, health and public health organizations, and social services agencies (Office of Adolescent Health 2019). For example, both of the school-based programs described in this supplement built engagement with community-based providers into their program model (Asheer et al 2020b, this issue; Aufrichtig et al. 2020, this issue). For New Heights, staff from community-based organizations were invited to conduct lunchtime workshops on topics such as contraception, parenting, and child development at least 3 times a week. Access to these providers during the school day enabled students to make connections to needed resources (Asheer et al. 2020b, this issue; Aufrichtig et al. 2020, this issue).

Despite the importance of partnerships for meeting the needs of expectant and parenting teens, service providers often operate in siloes and may not know about the services other providers in their area offer, leading to duplication (Purington et al. 2020a, this issue; Workman and Browder 2020, this issue). Systems-level approaches can facilitate better coordination of services for teen parents, for example, through convening regular meetings of service providers who serve similar teen parents (Purington et al. 2020a, this issue; Purington et al. 2020b, this issue; Workman and Browder 2020, this issue). In one school district in New York state, a program coordinator focused on developing connections among service providers. As more collaborative relationships were developed, staff reported it became easier to communicate, write referrals, and inform young parents of available services (Purington et al. 2020a, this issue). Similarly, stakeholders of a systems-level program in South Carolina perceived that a focus on improving collaboration among partners enhanced accessibility, reduced duplication of services, and improved collaboration among partner agencies (Workman and Browder 2020; this issue). As we will discuss later, social network analysis of the relationships between service providers may be a tool for helping providers understand existing partnerships and how those can be strengthened (Purington et al. 2020b, this issue). Overall, program implementers should consider how to develop partnerships to meet the complex needs of expectant and parenting teens.

## Hiring and Training Staff is Crucial for Strong Implementation

Staff are crucial to strong program implementation. For example, trusting relationships between staff and teen parents were identified as instrumental in successful participant retention (Egan et al. 2020, this issue). Articles in the supplement describe the importance of both hiring the right staff and providing training and technical assistance to support staff to work with teen parents, a population with extraordinary needs and challenges (Pinzon and Jones 2012; Savio Beers and Hollo 2009). Programs found it important to hire staff who were the right fit for the particular context; had strong connections in the community; had specific personality traits, skills, and knowledge; and were committed to “do whatever it takes” to support teen parents. For example, some programs aimed to hire staff with similar lived experience or prior experience with teen parents (Egan et al. 2020, this issue), and doing so was particularly important for staff working with young fathers (Niland and Selekman 2020, this issue). One successful approach to hiring staff was being “thoughtful about defining program requirements and expectations for staff early in the process, to help identify and recruit staff with the appropriate skills and personalities to fit the role” (Asheer et al. 2020b, this issue).

In addition to hiring the right staff, it was important to provide staff with training and support to work with teen parents. Some articles describe initial challenges with ensuring staff had enough training. For example, when implementing the statewide Adolescent Family Life Program-Positive Youth Development case management model, staff struggled to understand how to deliver the program day to day with mothers who were facing crisis (Asheer et al. 2020a, this issue). Similarly, staff struggled to understand and integrate content on reducing repeat teen births when implementing the Healthy Families Healthy Futures enhanced with Steps to Success home visiting program (Zief et al. 2020b, this issue). In both cases, the programs had to provide more training and supervision than staff initially expected (Asheer et al. 2020a, this issue; Pressfield et al. 2020, this issue; Zief et al. 2020b, this issue). Strategies for ensuring adequate staff training and support included setting up technical assistance calls to provide opportunities for sites to do more intentional, concrete planning about implementation (Asheer et al. 2020a, this issue); having program managers provide targeted one-on-one feedback based on observing program implementation as part of ongoing monitoring (Asheer et al. 2020b, this issue; Aufrichtig et al. 2020, this issue); and actively cultivating a culture of collaboration among program staff (Asheer et al. 2020b, this issue).

Programs also needed to provide training to staff serving fathers. For example, ongoing coaching and technical assistance from a male engagement expert and regular in-person or virtual peer-to-peer learning opportunities were provided to support case managers serving young fathers (McGirr et al. 2020, this issue). This training also focused on challenging any of staff’s negative stereotypes about young fathers (McGirr et al. 2020, this issue). In general, programs should expect that intensive support and guidance may be required to ensure staff can implement programs with fidelity, particularly when the program is new or still being developed (Pressfield et al. 2020, this issue).

## Recruiting and Engaging Teen Parents Requires Focused Attention

Recruiting and engaging expectant and parenting teens can be challenging because teen parents have many responsibilities and often experience barriers such as a lack of transportation or child care (Asheer et al. 2014). Multiple articles describe challenges recruiting and engaging teen parents. For example, in a program designed to support student parents in college, staff found it difficult to identify expectant and parenting students because colleges do not typically have lists of such students. As a result, some eligible students did not know about the program (Askelson et al. 2020, this issue). In another program, only 60% of youth randomized to the Healthy Families Healthy Steps supplemented with Steps to Success home visiting program had more than five visits within the first year even though visits were intended to be at least monthly (Zief et al. 2020b, this issue). Although engagement in the Adolescent Family Life Project-Positive Youth Development case management program was relatively high, barriers included transitions in clients’ lives, transportation issues, family and school commitments, and other overwhelming life circumstances. In addition, housing instability meant that families moved often, and it took time and effort to locate them again (Asheer et al. 2020a, this issue).

In response to these challenges, PAF grantees identified strategies to enhance recruitment and engagement, including having a program champion to promote activities, offering teen parents social activities and support groups, building strong staff-participant relationships, providing concrete supports to teen parents, and using technology. First, having a program champion (faculty or staff) within colleges helped to identify and recruit participants. Program administrators reported the program champion’s recruitment via word of mouth and his or her promotion of activities/services were most successful in reaching potential PAF participants (Askelson et al. 2020, this issue). Second, social-emotional supports such as social activities and support groups were frequently mentioned by participants as reasons for remaining in a case management program (Egan et al. 2020, this issue). Third, as previously mentioned, strong staff relationships can support engagement; one study found stronger engagement in programs that used a centralized staffing model with youth assigned to a youth worker, rather than in a program with a more diffuse staffing model (Egan et al. 2020, this issue). Fourth, providing concrete supports, such as child care and transportation, helped address barriers to parents’ participation (Egan et al. 2020, this issue). Finally, using information and communication technology for case management helped case workers reach difficult-to-reach clients by having multiple communication methods (Kang et al. 2020, this issue). For example, case workers could use Facebook if clients’ phones were disconnected. Technology also provided flexibility to address transportation and time barriers. However, case workers believed the technology limited their ability to build rapport, and they expressed the need for face-to-face and telephone contact in addition to using text, email, and social media.

Recruiting and engaging teen fathers is particularly challenging, in part because they are harder to identify. For example, although a school-based program like New Heights attracted 75% of all teen mothers in their schools (Asheer et al. 2017b), just a fraction of participants were fathers. The school-based coordinators cited identifying fathers as a key issue. Fathers can also be disconnected from services because they are often not the custodial parent (Niland and Selekman 2020, this issue). Again, PAF grantees described multiple strategies for recruiting and engaging young fathers, including recruiting from places that fathers frequent and by word of mouth, providing programming and activities that were welcoming to fathers, connecting fathers to other young fathers, valuing fathers’ feedback, and setting minimum caseloads for serving fathers. First, effective recruitment strategies included recruiting from places that fathers naturally frequent such as gyms, barbershops, and community centers (Niland and Selekman 2020, this issue) and using word of mouth from other fathers (McGirr et al. 2020, this issue). Second, programs created environments that were welcoming to fathers. For example, they featured pictures of men on recruitment materials (McGirr et al. 2020, this issue; Niland and Selekman 2020, this issue) and provided father-focused activities such as game nights and video games (Niland and Selekman 2020, this issue). Third, programs connected young fathers to one another, and young fathers reported they enjoyed the opportunity to connect with other young fathers (McGirr et al. 2020, this issue). Fourth, program staff made decisions based on young fathers’ feedback and fathers and case managers believed this contributed to retaining these fathers (McGirr et al. 2020, this issue). Finally, setting minimum caseload expectations for serving fathers helped ensure fathers stayed top of mind (McGirr et al. 2020, this issue). In general, programs need to pay focused attention to recruiting and engaging teen parents, particularly fathers.

## Sustainability

In the context of limited funding and the substantial needs of teen parents, organizations must focus on sustainability to maintain services after grant funding ends (Seifer 2006). The PAF grant included expectations for sustainability planning, and the Office of Populations Affairs provided sustainability planning resources and technical assistance (Asheer et al. 2017a; Warner et al. 2020, this issue; Office of Adolescent Health 2017). Grantees found it useful to begin planning for sustainability early to sustain aspects of their program after PAF funding ended (Warner et al. 2020, this issue). Similarly, a case study of college-based student parent centers that were sustained after PAF funding ended showed the grantee set up a sustainability committee that prepared a sustainability plan early in the grant process (Amenumey et al. 2020, this issue).

Communicating regularly with key stakeholders, building partnerships, and other sustainability activities helped grantees to identify new funding sources, keep programming relevant, and build support for their programs (Amenumey et al. 2020, this issue; Warner et al. 2020, this issue). Indeed, a sustainability committee set up to support student parent centers included key stakeholders from institutions of higher education, local public health agencies, and state agencies (Amenumey et al. 2020, this issue). In addition, representatives of the student support centers were trained on communicating organizational success and delivered these messages to foundations, state legislators, and university leadership (Amenumey et al. 2020, this issue). Building partnerships with other organizations also supported sustainability (Warner et al. 2020, this issue). Two grantees joined local coalitions, which enabled organizations to work together to strengthen funding applications, as opposed to competing with one another (Warner et al. 2020, this issue). Similarly, two articles about systems-level approaches describe how improved partnerships among community providers helped communities (1) gain additional grant funds (Workman and Browder 2020, this issue) and (2) institutionalize policies and procedures for service coordination that could last even after funding ended (Purington 2020a, this issue). Other important contributors to sustainability involved diversifying funding sources, including using federal, state, private, and in-kind support to support different program components (Warner et al. 2020, this issue). In addition, implementing evidence-based interventions allowed grantees to access additional funding streams and cite evidence of effectiveness when presenting information to stakeholders (Warner et al. 2020, this issue). Finally, collecting and sharing data on program success provided concrete information to make the case for the importance of student parent centers (Amenumey et al. 2020, this issue). Early on, programs should pay attention to sustainability and develop strategies to maintain funding and partnerships to continue to serve teen parents.

## Innovative Qualitative and Quantitative Methods can be Used to Evaluate Programs

The studies in this supplement use a range of qualitative and quantitative methods to provide insight into serving expectant and parenting teens. In addition to more traditional research methods, some studies use innovative methods such as presenting Bayesian impact estimates alongside traditional impact estimates (Zief et al. 2020b, this issue), using a quasi-experimental design based on administrative data (Zief et al. 2020a, this issue), conducting “ripple effect mapping” to gather stakeholder perceptions of program benefits (Workman and Browder 2020, this issue), and using social network analysis to understand partner relationships (Purington et al. 2020b). Three articles specifically describe how to implement these innovative methods and can contribute to the toolkit for evaluators of programs to support expectant and parenting teens.

The evaluation of the New Heights school-based program used a rigorous difference-in-differences design based on administrative data to determine effects on teen mothers’ educational outcomes (Zief et al. 2020a, this issue). The article in this supplement shares lessons learned from implementing this rigorous quasi-experimental method, including the importance of having a well-specified logic model to identify potential outcomes that can be measured with administrative data, getting staff buy-in to enhance understanding of program implementation, and building strong partnerships between program staff and evaluators (Zief et al. 2020a, this issue). Through using extant administrative data, maternal and child health programs may be able to obtain rigorous evaluations at reasonable cost (Zief et al. 2020a, this issue).

Participatory research methods can involve community members in analyzing and reflecting on information generated by the research process. Two articles present examples of using participatory methods to evaluate systems-level approaches to serving teen parents, which may be particularly challenging to evaluate because of their complexity. One article used ripple effect mapping to identify stakeholders’ perceptions of success through a participatory process (Workman and Browder 2020, this issue; Chazdon et al. 2017; Kollock et al. 2012; Olfert et al. 2018). Program stakeholders were engaged in meetings to describe and visually map their perceptions of the effects of a systems-level approach to coordinating services for teen parents in South Carolina. The method allowed stakeholders to describe their perspectives on what the program achieved in communities, explain different perspectives, and reflect together to develop a shared narrative through a systematic, facilitated process. A different participatory approach used stakeholder reflections on a social network analysis of providers in the community to help understand the relationships between partners, including the number and diversity of organizations connected to one another and the intensity of those relationships (Purington et al. 2020a, this issue; Varda et al. 2008). This study used a survey and analysis tool to map and describe the local network of service providers serving teen parents. The authors then used a participatory process in which community partners met to reflect back on these maps and develop next steps for increasing collaboration. Each community developed specific, actionable steps to strengthen organizational networks. Evaluators of programs serving expectant and parenting teens should consider methods from the full research toolkit, including both qualitative and quantitative methods as well as more innovative, participatory approaches.

## Summary

This supplement makes important contributions to both research and practice related to serving teen parents. Two impact studies, one of which was a replication study, and a systematic review, which included other recent studies funded by the U.S. Department of Health and Human Services in addition to a broader range of studies, illustrate that there are effective programs for promoting positive outcomes for teen parents (Zief et al. 2020a, b, this issue; Harding et al. 2020, this issue). Researchers observed impacts on key aspects of teen parents’ self-sufficiency: educational progress, use of contraception, and reductions in repeat pregnancies or births. Effective programs can use different approaches to serving teen parents.

In addition, the supplement articles demonstrate five key lessons learned from implementation, including allowing some flexibility in implementation while clearly outlining core programmatic components, using partnerships to meet the multifaceted needs of young parents, hiring the right staff and extensively training them, using strategies for engaging and recruiting teen parents, and planning for sustainability early. First, outlining core program components but allowing flexibility can assist programs to meet the complex needs of teen parents. Program developers and supervisors may wish to incorporate guidance on how program implementers can be flexible while maintaining fidelity to the core components. Second, partnerships are needed to support the multifaceted needs of teen parents. Program implementers should consider how to develop strong partnerships to meet the complex needs of expectant and parenting teens. Third, staff are crucial for strong program implementation. Programs should work to hire the right staff to serve teen parents, including teen fathers; they can do this by clearly defining expectations for staff. In addition, programs should expect that intensive support and guidance may be required to ensure program staff implement programs with fidelity, particularly when the program is new. Fourth, recruiting and engaging teens is challenging. Programs must focus attention on recruiting and engaging teen parents, particularly fathers. Strategies that may be effective to support recruitment and engagement include having a program champion to promote activities, offering teen parents social activities and support groups, building strong staff-participant relationships, providing concrete supports to teen parents, and using technology. Fifth, early and strategic planning for sustainability can help programs support teen parents over the long term.

In addition to adding to the knowledge base on what works to support teen parents and lessons learned in serving them, the supplement also illustrates how a range of qualitative and quantitative methods can provide insight into serving teen parents. Using administrative data can help programs rigorously evaluate their outcomes in a more cost-effective way. In addition, more innovative, participatory approaches can provide useful tools for evaluating complex initiatives. Evaluators of programs serving pregnant and parenting teens should consider methods from the full research toolkit.

## Next Steps for Future Research

Although these studies provide important insight into serving teen parents, additional research is needed. Only two studies provide rigorous evidence of program effectiveness. We need more rigorous research, including on what works to support groups who have received less attention such as fathers, college-age parents, and parents experiencing intimate partner violence. Programs are often small, which is a challenge for rigorous evaluation, but new Bayesian methods can be helpful for interpreting findings from small-scale evaluations. Next, program components research that examines how specific components contribute to outcomes would be useful because programs are often multi-component. We also need more systematic examination of partnerships to serve teen parents. Social network analysis may be a promising tool for understanding how partnerships strengthen over the course of program implementation, but rigorous research is needed to determine whether strengthening partnerships actually improves service delivery and teen parents’ outcomes. Finally, these articles provide suggestive evidence of how to recruit and engage teen parents, including fathers, but more rigorous research using rapid cycle learning methods to trial such tactics could help programs understand the most effective recruitment and engagement strategies.

## Footnotes

1 This article summarizes across other articles so it is not based upon clinical study or patient data.
